# Characterizing fall risk factors in Belgian older adults through machine learning: a data-driven approach

**DOI:** 10.1186/s12889-022-14694-5

**Published:** 2022-11-29

**Authors:** Elke Lathouwers, Arnau Dillen, María Alejandra Díaz, Bruno Tassignon, Jo Verschueren, Dominique Verté, Nico De Witte, Kevin De Pauw

**Affiliations:** 1 Human Physiology and Sports Physiotherapy Research Group, Vrije Universiteit Brussel, 1050 Brussels, Belgium; 2 Brussels Human Robotic Research Center (BruBotics), Vrije Universiteit Brussel, 1050 Brussels, Belgium; 3 Faculty of Psychology and Educational Sciences, Vrije Universiteit Brussel, Pleinlaan 2, 1050 Brussels, Belgium; 4 Gerontology and Frailty in Ageing (FRIA) research department, Vrije Universiteit Brussel, Laarbeeklaan 103, 1090 Brussels, Belgium

**Keywords:** Fall incidence, Older adults, Risk factors, Machine learning

## Abstract

**Background:**

Falls are a major problem associated with ageing. Yet, fall-risk classification models identifying older adults at risk are lacking. Current screening tools show limited predictive validity to differentiate between a low- and high-risk of falling.

**Objective:**

This study aims at identifying risk factors associated with higher risk of falling by means of a quality-of-life questionnaire incorporating biological, behavioural, environmental and socio-economic factors. These insights can aid the development of a fall-risk classification algorithm identifying community-dwelling older adults at risk of falling.

**Methods:**

The questionnaire was developed by the Belgian Ageing Studies research group of the Vrije Universiteit Brussel and administered to 82,580 older adults for a detailed analysis of risk factors linked to the fall incidence data. Based on previously known risk factors, 139 questions were selected from the questionnaire to include in this study. Included questions were encoded, missing values were dropped, and multicollinearity was assessed. A random forest classifier that learns to predict falls was trained to investigate the importance of each individual feature.

**Results:**

Twenty-four questions were included in the classification-model. Based on the output of the model all factors were associated with the risk of falling of which two were biological risk factors, eight behavioural, 11 socioeconomic and three environmental risk factors. Each of these variables contributed between 4.5 and 6.5% to explaining the risk of falling.

**Conclusion:**

The present study identified 24 fall risk factors using machine learning techniques to identify older adults at high risk of falling. Maintaining a mental, physical and socially active lifestyle, reducing vulnerability and feeling satisfied with the living situation contributes to reducing the risk of falling. Further research is warranted to establish an easy-to-use screening tool to be applied in daily practice.

**Supplementary Information:**

The online version contains supplementary material available at 10.1186/s12889-022-14694-5.

## Introduction

Community-dwelling older adults frequently report falling. Roughly 30% of the older adult population falls at least once a year and about 15% at least twice a year [1]. Falls are one of the main problems associated with ageing and are among the major causes of injuries and mortality in older adults. This induces a spectrum of adverse health outcomes such as decreased quality of life and functional independence [2, 3]. Regarding falls requiring health care, Belgium has an incidence ratio of 19,634 per 100,000 falls, which is amongst the highest in Western Europe and entails high medical costs [4]. Furthermore, approximately 19% of the Belgian population is aged over 65 and this number is expected to increase to 25% by 2070 [5]. Belgium’s current healthcare costs combined with the socioeconomic challenge of supporting the health management of an increasingly ageing population, will impose a burden on our society. Such a tendency will not be unique to Belgium but is also expected to occur globally [6]. Therefore, preventing fall incidence is essential to avoid over-burdening healthcare systems.

Identifying risk factors is critical in developing fall prevention strategies to minimise the number of falls in older adults. According to the World Health Organization, risk factors can be classified into biological, behavioural, environmental and socio-economic categories [7]. Sedentary lifestyle, lack of physical activity, fearful behaviour, previously fallen and polypharmacy are known to be the behavioural risk factors [8–11]. Socioeconomic risk factors include age, household type, marital status, education level, current employment status, past career, annual income, personal wealth, number of children and relationship satisfaction [12]. Biological risk factors include, among others, the age-related deterioration in physical abilities like sarcopenia and decrease in balance along with impaired vision, hearing and cognitive decline [13–17]. Furthermore, sex, overall health status and psychological state of mind (e.g., presence of a depression) also encompass this category [13, 17–20]. The last category refers to the environmental risk factors, such as poor housing conditions, inadequate lighting or slippery floors provoking hazards [19, 21, 22]. Falls tend to stem from a sophisticated cluster of risk factors, which cumulatively leads to a person’s inability to retain or retrieve stability and balance [2, 23]. For example, the degree of frailty is an overarching risk factor of falling [24]. Frailty can be defined as a clinically identifiable condition of heightened vulnerability that results from age-related declines in reserves and functions in several physiological systems, leading towards reduced ability to cope with stressors [25]. It incorporates social, emotional, physical, psychological and cognitive components as well as environmental elements [25]. Moreover, the degree of frailty is also dependent on socioeconomic status. A higher socioeconomic status tends to coincide with a reduced likelihood of frailty [26]. Interactions of risk factors arise not only across categories, but also within each one. For instance, within the category of behavioural risk factors, the combination of depression and malnutrition has been demonstrated to increment fall risk [20]. Due to the jumble of interactions between risk factors, making it an extremely complex ensemble, these aforementioned factors reiterate the importance of multifactorial bio-psychophysiological tailored prevention programmes to reduce the risk of falling.

Predicting medical outcomes with machine learning (ML) to improve preventative or curative strategies was successfully achieved in multiple contexts [27–29]. This lets us assume that fall risk could also be predicted with similar methods. Nevertheless, the success of developing such a model is highly dependent on the quality and amount of the data available [27, 30]. ML is a data-driven subfield of artificial intelligence where a statistical model is built from a set of so-called training examples [27, 30]. Building these models can either consist of finding the optimal set of parameters that best fit the data or of using similar instances of input to determine the output [27, 30]. Up to now, fall-risk classification models for screening purposes based on the aforementioned risk factors are lacking [31, 32]. Also, current screening tools show limited predictive validity to differentiate between low- and high-risk fallers [33, 34]. In addition, research combining all categories of risk factors for falls appears to be scanty. It is noteworthy that a substantial number of recent studies have been conducted on Asian populations [8, 9, 12, 14, 16, 17, 20, 21, 26, 35]. This might be explained by the fact that the older adult population living within Asia is rapidly increasing [23]. Since, it has been documented that racial and geographical differences have an influence on the fall risk and incidence [36], and older adults living in rural areas report a higher fall incidence compared to older adults living in an urban area [35], it is questionable to what extent these conclusions can be transferred to community-dwelling older adults living in Western Europe. Therefore, the purpose of this study is to identify risk factors contributing to an incremented fall incidence by means of a quality-of-life questionnaire incorporating biological, behavioural, environmental and socio-economic factors. These insights can provide healthcare providers with new perspectives into the most prominent fall risk factors. Furthermore, these insights can contribute to developing a fall-risk classification algorithm that identifies community-dwelling older adults at higher risk of falling so that those identified at risk can timely be provided with adequate fall-prevention programmes.

## Methods

The large-scale availability of data on fall incidence and associated risk factors allows for a more advanced statistical analysis to identify the most critical risk factors. In this study, a questionnaire questionnaire assessing needs and quality of life, developed by Belgian Ageing Studies (Vrije Universiteit Brussel) [37] and administered to 82,580 older adults (2004–2020) was used for a detailed analysis of risk factors linked to the fall incidence data. The data was gathered by means of stratified random sampling (sex and age) in participating municipalities drawn from census data of community-dwelling older adults aged 60 and over living in Belgium [37]. Based on previously known risk factors, 139 questions were selected from the questionnaire to include in this study. The experimental design was approved by the medical Ethics Committee of the university hospital and Vrije Universiteit Brussel (B.U.N. 143,201,111,521).

Risk factors were analysed with ML techniques, which are presented below, learning to predict fall risk based on the other factors in the questionnaire. By analysing how the trained ML models make decisions, insights can be gained regarding risk factors' individual and combined contributions. ML consists of training a statistical model to predict a class or a value, given a set of training examples [27, 30]. The end-goal is to develop a model that can predict outputs for unseen inputs [27, 30]. The inputs of the model are the answers to the different questions and the output is whether a person reported falling.

### Data pre-processing

Before training the ML models, the data was pre-processed to deal with missing values and noisy features. We excluded questions with more than 30% missing data. Participants that had remaining missing data on the inputs of the model were also dropped. Then, we encoded questions with categorical factors and introduced an ordering to categories. The correct ordering for ordinal questions with the original categories was set for the questions on the variables loneliness, physical exertion, mental activity, income, housing issues, feeling unsafe, physical vulnerability, psychological vulnerability, social vulnerability, environmental vulnerability, age category. Sex, civil status, number of (grand)children, homeownership and the home type, remained unchanged. We also converted categories to more high-level representations to reduce the number of categories where this was relevant as high cardinality can induce significant noise in most statistical analyses. For example, we converted the postal code to an urbanisation category (i.e. surrounding density) based on the population density. The variables surrounding density, housing change, organisation of the neighbourhood, level of education, mode of transportation, having help available, physical activity and help required were recoded to reduce the number of categories and questions.

**Fig. 1:**
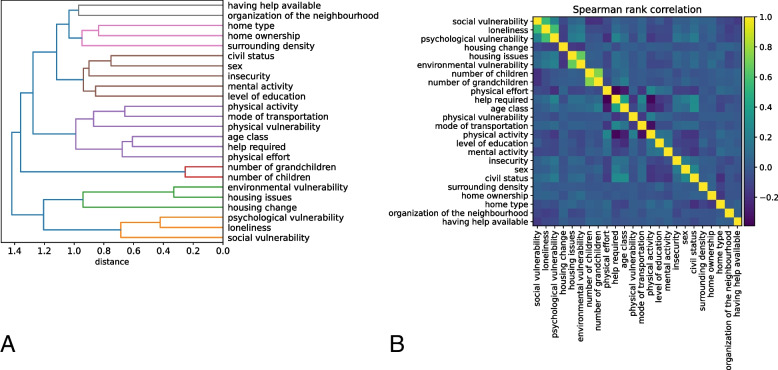
Clustering of the variables contributing to falls. **A** visualises the clustering between the included features based on the distance matrix applying the Ward’s linkage method. **B** depicts the cross-correlation of the included features using a spearman rank correlation

Next, multicollinearity was assessed between input features through clustering (Fig. 1). All variables from the questionnaire were retained for constructing the decision tree (i.e. social vulnerability, loneliness, psychological vulnerability, housing change, housing issues, environmental vulnerability, number of children and grandchildren, physical effort, help required, age class, physical vulnerability, mode of transportation, physical activity, level of education, mental activity, insecurity, sex, civil status, surrounding density, home ownership, home type, organisation of the neighbourhood, and having help available). A distance matrix was computed between all remaining features by means of the inverse of cross correlation (i.e., Spearman rank), and then used the distance matrix to cluster the features by applying the Ward’s linkage method [38]. In total, we excluded 84 questions based on the amount of missing data and questions included in high-level constructs. This resulted in 24 input features and 33,346 remaining entries. An overview of the questions included in the 24 input features is provided in Supplementary Materials S1.

### Random forest model building

Subsequently, a random forest classifier that learns to predict the number of times a person would fall within the coming year was trained to investigate the importance of each individual feature. Random Forests are a type of ML model that combines an ensemble of decision trees trained on a subset of data by using only a subset of the available features [39]. We used extremely randomized trees (i.e., Extratrees) for our analyses, a more sophisticated variant of random forests [40]. The random forest approach was chosen for its explainability and ability to deal with categorical variables [27, 30]. Indeed, since random forests consist of multiple decision trees, these trees can be visualized to investigate the decision process in each tree, which is interpretable [27, 30]. The classification performance of our models was estimated by tenfold cross-validation [41] to ensure that the model does not overfit. We chose 10 folds (i.e. 90% data used for training and 10% left out for testing) to ensure that the resulting accuracy represents performance on previously unseen test data [27, 30].

After training a random forest, the contribution of individual features was extracted by observing information gain. When training a decision tree, the split (i.e., values or categories that determine which branch to follow) is determined by looking at information gain [42]. The split that results in the highest information gain is then selected and the process is repeated until a stopping criterium is reached (e.g., maximum depth of the tree). By averaging the information gain for each feature upon its use in a decision tree, we can rank the features. The higher the average information gain, the more important the feature is considered.

Our Random Forest classifier consisted of 500 individual estimators and used entropy as the criterion to split nodes in the decision trees. The number of 500 trees was chosen to ensure that each input feature was used in multiple decision trees, as we have no control over which features are selected due to the random feature selection of Extratrees. To ensure that model initialization does not influence the results, model training and feature importance estimation were repeated 100 times [27, 30]. This number was chosen to ensure a high statistical power that can compensate for randomization effects. Results are provided as averages over each of these iterations [27, 30].

The analyses were performed with the Python programming language, using the SciPy library to compute statistics and perform statistical tests [43, 44]. We conducted tabular data manipulations with the NumPy and Pandas libraries and generated figures with the Matplotlib software package [45–47]. Finally, we used scikit-learn to construct and evaluate ML models [48].

### Participants’ characteristics

The ML model is based on the input of 33,346 community-dwelling older adults of which 51.4% were female and 49.6% were male. The mean age and standard deviation amounted 71 ± 8 years. Among, 49.6% were aged between 60 and 69, 34.4% between 70 and 79 and the remaining 16% were aged over 80 years old.

## Results

**Fig. 2:**
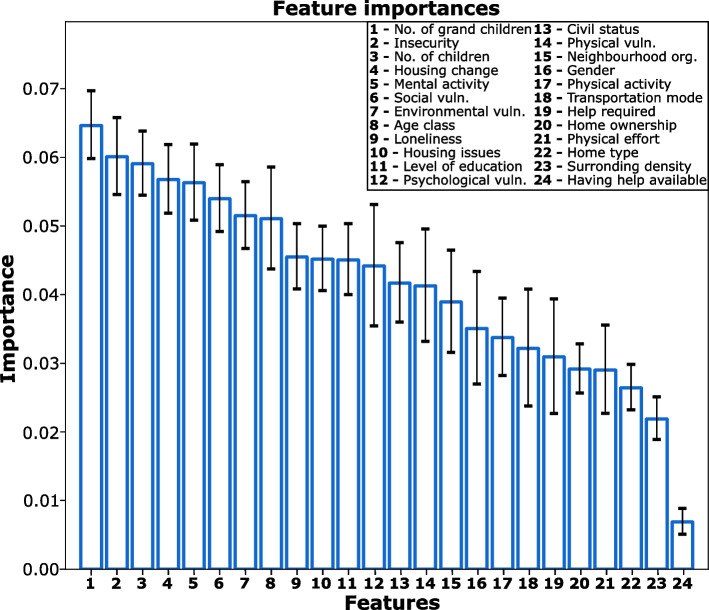
Feature importance determined from the mean decrease in impurity when building the decision trees of the random forest

Based on a random forest classifier, the contribution of each of the 24 individual features within the decision tree was determined by means of “individual feature importance”. The model reached an average of 73% accuracy. The mean importance of each feature and its standard deviation over 100 iterations are visualised in Fig. 2.

Number of grandchildren is the variable that contribute more to the risk of falls with a mean contribution and standard deviation of 6.5 ± 0.5%, followed by insecurity (6.0 ± 0.6%), number of children (5.9 ± 0.5%), housing change (5.7 ± 0.5%), mental activity (5.6 ± 0.5%), social vulnerability (5.4 ± 0.5%), environmental vulnerability (5.2 ± 0.5%), age class (5.1 ± 0.7%), loneliness (4.5 ± 0.5%) and housing issues (4.5 ± 0.5%). Level of education contributed 4.5 ± 0.5%, psychological vulnerability 4.4 ± 0.5%, civil status 4.2 ± 0.6%, physical vulnerability 4.1 ± 0.8%, organisation of the neighbourhood 3.9 ± 0.7%, sex 3.5 ± 0.8%, physical activity 3.4 ± 0.5%, mode of transportation 3.2 ± 0.8%, help required 3.1 ± 0.8% and home ownership contributed 2.9 ± 0.3%. Physical effort explained 2.9 ± 0.6% of the falls, home type 2.6 ± 0.3%, surrounding density 2.2 ± 0.3% and having help available contributed marginally with 0.7 ± 0.2%.

## Discussion

This study aimed to identify risk factors contributing to an incremented fall incidence through a questionnaire which could aid the development of a fall-risk classification algorithm identifying community-dwelling older adults at higher risk of falling. To the best of our knowledge, this is the first study using artificial intelligence to attempt predicting falls in older adults based on questionnaires incorporating biological, behavioural, environmental and socioeconomic- risk factors. Due to the multifactorial aspect of the results the interpretation should be done with caution and due to the unique approach, the comparison of our results to existing literature cannot be performed.

Our findings showed 24 variables contributed to predicting the occurrence of a fall. Among these, two were biological risk factors (loneliness, sex), eight were behavioural (i.e. physical vulnerability, physical effort, physical activity, mental activity, help required, having help available, mode of transportation and psychological vulnerability), eleven were socioeconomic (i.e. age class, level of education, civil status, surrounding density, homeownership, home type, number of children and grandchildren, insecurity, organisation of the neighbourhood and social vulnerability) and three were environmental risk factors (i.e. housing issues, housing change and environmental vulnerability). The majority of these factors mentioned above were positively correlated with the risk of falling. Only mental activity, having help available, level of education, the number of children and grandchildren, and the neighbourhood's organisation were negatively correlated.

In general, our results imply that apart from the intrinsic factors of ageing and sex, maintaining a mental, physical and socially active lifestyle, reducing an individuals’ vulnerability, maintaining interaction with others (e.g., family, friends, neighbours) and feeling satisfied with the living situation contributes to reducing the risk of falling. These results appear to be consistent with the fall risk factors already investigated and the risk factors for frailty presented in the dynamic D-scope model [2, 23, 49–51]. Frailty is highly associated with the risk of falling and can be defined as a clinically identifiable condition of heightened vulnerability that results from age-related declines in reserves and functions in several physiological systems, leading towards reduced ability to cope with stressors [24, 25, 52, 53]. The D-scope model illustrates that the risk factors for frailty contain a balancing state between individual, environment-related and macro-level factors on the one hand and between cognitive, environmental, physical, psychosocial and social health factors on the other hand. Those interactions between risk factors for frailty seem to be in accordance with the interactions of risk factors found in our current study in the context of fall risk and fall prevention. It is well established that fall risk factors include biological, behavioural, environmental and socioeconomic factors as well as overarching factors creating interaction with each factor, such as frailty [2, 7, 23]. As a result of the intertwining of fall-risk factors, disentangling the network and providing a unifying risk profile is not straightforward [24, 52, 54]. Consequently, we excluded umbrella risk factors such as vulnerability from our analysis to simplify the model. Despite this, we found that the included variables were correlated with each other, suggesting that predictive models could be simplified by incorporating profiles such as an activity profile comprising a combination of means of transportation, physical exertion and degree of physical activity to establish a robust risk profile (Fig. 1).

For our analysis, we had to exclude variables known to significantly contribute to falls to reduce the volume of missing data, such as weight loss, polypharmacy, impaired vision and hearing, and cognitive decline [54–56]. The amount of missing data that initially resulted in zero complete data entries was attributable to the questionnaire increasing in size during the data collection process and to older adults incorrectly answering questions. By excluding variables with more than 30% missing data and dropping entries with remaining missing data, we could attain 33,346 out of 82,580 complete data entries. As a result, findings must be interpreted carefully.

A random forest classifier with feature importance analysis was used to attain a fall prediction model. This model reached an average of 73% accuracy when using our data engineering and parameter settings. However, if predictive accuracy is the primary goal, the current model might not be the most optimal. On the one hand, the chosen parameter settings were based on manual trial and error without performing an exhaustive search of the parameter space. On the other hand, the choice of the model itself might not be the best. More advanced methods, such as deep learning, could potentially result in better accuracy at the cost of decreased explainability [27, 30]. However, applying those methods to the current dataset goes beyond the scope of the current study.

This study illustrates the possibility of creating a decision tree through machine learning techniques. In order to obtain a usable screening tool for correctly identifying people at risk of falling, future research must focus on creating a robust and feasible decision tree that incorporates the relationships between the various factors to the best extent with large and clean data samples. On top of this, the application of machine learning should be enhanced. Possible enhancement could be made by further investigating the data engineering and multicollinearity of the different factors [27, 30]. As we identified features contributing to falls, other ML methods like support vector machines or neural networks could be used to improve accuracy [27, 30]. Also, accuracy could be increased by fine-tuning the Extratrees model's hyperparameters by a parameter search [27, 30]. Further elaboration will require close collaboration between gerontologists, data scientists and other care providers who are closely involved in this line of research.

## Conclusion

The present study identified 24 fall risk factors. It illustrated the possibility of creating a decision tree through machine learning techniques to predict falls in community-dwelling older adults based on a questionnaire. Future research is warranted to establish a more robust screening tool for use in daily practice, correctly identifying people at risk of falling and integrating the relationships between different factors using clean data.

## Supplementary Information

**Additional file 1.**

## Data Availability

The data underlying this article were provided by the Belgian Ageing Studies research group of the Vrije Universiteit Brussel and cannot be shared publicly due to ethical reasons but are available from Nico De Witte (Nico.De.Witte@vub.be) on reasonable request**.**

## Abbreviations

ML: Machine learning
