# Supplementary material for: Characterizing fall risk factors in Belgian older adults through machine learning: a data-driven approach
Source: BMC Public Health. 2022 Nov 29;22:2210. doi: 10.1186/s12889-022-14694-5 (PMC9707258; doi:10.1186/s12889-022-14694-5)
Supplement: Supplementary file 1 — Additional file 1. [file 12889_2022_14694_MOESM1_ESM.pdf]

Supplementary Materials

Table S. 1: Questions incorporated in final input features

Features	Questions incorporated in input features
Social vulnerability	To what extent do you agree with the following statements? (categorical answers)<br>• There are enough people I can fall back on in case of trouble<br>• I know many people I can completely rely on.<br>• There are enough people with whom I feel connected<br>How satisfied are you with the contact with the following people? (categorical answers)
Loneliness	To what extent do you agree with the following statements? (categorical answers)<br>• I experience an emptiness around me.<br>• There are enough people I can fall back on in case of trouble<br>• I know many people I can completely rely on.<br>• There are enough people with whom I feel connected<br>• I miss people around me.<br>• I often feel abandoned.
Psychological vulnerability	Considering the past few weeks, to what extent do you agree with the following statements? (categorical answers)<br>• I sleep badly and often lie awake due to headaches<br>• I feel unhappy or depressed<br>• I feel I am losing my self-confidence<br>• I feel that I cannot cope with the problems<br>• I feel like I am constantly under stress<br>• I feel like I am not worth anything anymore
Housing change	How do you stand towards the following options? (categorical answers)<br>• Leaving your property as it is<br>• Adapting your home to your needs<br>• Moving to an adapted home<br>• Moving to a retirement home/ rest and care home/ residential care centre<br>• Living with children<br>• Living with several elderly people, with separate living quarters<br>• Moving to service flats/assisted living
Housing issues	Which statements apply to your property? (dichotomous answers)<br>• Property is too big<br>• Property is too small<br>• Property is in poor condition/is poorly maintained<br>• I have to climb stairs to enter the property<br>• The thresholds are too high (inside or outside the property)<br>• There are stairs in the property<br>• Property is too expensive<br>• Property is prone to burglary<br>• Property is not very comfortable<br>• Property is too noisy (poor sound insulation)<br>• Property is difficult to heat<br>• Property is not comfortable enough<br>• I don't like the neighbourhood<br>• Distance to amenities (e.g. shop, bank, ...) is too big<br>• Distance to children is too big
Environmental vulnerability	Which statements apply to your property? (dichotomous answers)<br>• Property is in poor condition/is poorly maintained<br>• Property is not very comfortable<br>• Property is difficult to heat<br>• Property is not comfortable enough<br>• I don't like the neighbourhood
Number of children	How many own and/or adopted children, alive, do you have?
Number of grandchildren	How many grandchildren, alive, do you have?
Physical effort & Physical vulnerability	Has your health condition limited you in the following activities and, if so, for how long?<br>• Very strenuous activities such as lifting heavy objects,<br>• Less strenuous activities (e.g. carrying groceries)<br>• Walking up a hill or up some stairs<br>• Bending, lifting or stooping<br>• Walking around a block<br>• Eating, dressing, showering, taking a bath or going to the toilet<br>• Household chores<br>• Social activities (e.g. visiting friends or family)
Help required	Do you need help with: (Dichotomous answers)<br>• Your personal care<br>• Your household<br>• Your personal movements
Age class	How old are you?

Mode of transportation	How do you transport yourself? (Categorical answers)<br>• On foot<br>• On foot with aids (walking stick, walker)<br>• Trolley, mobility scooter<br>• By car<br>• Bicycle<br>• Bus/ tram/ metro<br>• By train<br>• Taxi<br>• Call bus<br>• Transport organised by private or public<br>• Services (e.g. mobility centre,volunteers)
Physical activity	How often do you practice the following activities? (Categorical answers)<br>• Walking or cycling<br>• Other sports<br>• Playing cards or board games<br>• Acting, folk dancing, choir<br>• Crafts or handicrafts<br>• Going to a café or eating out (including brasserie and tearoom)<br>• Travelling and taking trips<br>• Gardening<br>• Reading books<br>• Taking an education or course<br>• Home repairs<br>• Shopping for pleasure<br>• Going to sporting event<br>• Going to library
Level of education	What is the highest education you have completed? (Categorical answer)
Mental activity	To what extent do you agree with the following statements? (Categorical answers)<br>• I have difficulty remembering what happened recently.<br>• I experience difficulties learning new things.<br>• I experience difficulties managing money matters<br>• I have difficulty following the story in a book or on television.
Insecurity	To what extent do you agree with the following statements? (Categorical answers)<br>• It is too unsafe nowadays to go out on the streets at night<br>• It is too unsafe nowadays to leave children alone on the street<br>• I don't go out much alone nowadays because I'm afraid of being mugged and robbed<br>• You have to be extra careful in the street at night<br>• In the last 10 years the streets have become more unsafe<br>• In the evening and at night I don't open the door when the doorbell rings<br>• In this day and age, an alarm system is not a luxury<br>• When I go on holiday, I dare not leave my house unguarded
Sex	Sex? (Categorical answer)
Civil status	Civil status? (Categorical answer)
Surrounding density	Postal code & Neighbourhood?
Home ownership	Which statement regarding your property applies to you?<br>• I am an owner<br>• I am a tenant (social housing)<br>• I am a tenant (private market)<br>• None of the above
Home type	What is your current form of housing? (Categorical answer)
Organisation of the neighbourhood	Do you think there is enough organised in your neighbourhood for people, over 60? (Categorical answers)
Having help available	Suppose you would not be able to do the activities you usually do in the household for a certain period of time, to whom could you turn? (Dichotomous answers)<br>• Spouse/partner<br>• Daughter<br>• Son<br>• Daughter-in-law<br>• Son-in-law<br>• Grandchild or great-grandchild<br>• Sister or brother-in-law (sister-in-law/brother-in-law)<br>• Other family member<br>• Neighbour<br>• Friend/ acquaintance<br>• To no one
